# Applicability of an immersive virtual reality system for assessing route learning in older adults

**DOI:** 10.1590/1980-5764-DN-2021-0006

**Published:** 2022-04-29

**Authors:** Michelle Didone dos Santos, Juliana Magalhães da Silva, Raquel Quimas Molina da Costa, Larissa Alamino Pereira de Viveiro, Emerson Galves Moretto, Roseli de Deus Lopes, Sonia Maria Dozzi Brucki, José Eduardo Pompeu

**Affiliations:** 1 Universidade de São Paulo, Faculdade de Medicina, Departamento de Fonoaudiologia, e Terapia Ocupacional, São Paulo SP, Brazil.; 2 Universidade de São Paulo, Faculdade de Medicina, Departamento de Neurologia, São Paulo SP, Brazil.; 3 Universidade de São Paulo, Faculdade Politécnica, Departamento de Engenharia, São Paulo SP, Brazil.

**Keywords:** Orientation, Spatial, Aged, Virtual Reality, Technology, Validation Study, Orientação Espacial, Idoso, Realidade Virtual, Tecnologia, Estudo de Validação

## Abstract

Spatial orientation is defined as the ability to find one’s way around an environment, follow familiar routes, recognize places, and learn new routes. Spatial disorientation is one of the early symptoms of Alzheimer’s disease (AD), and traditional cognitive evaluation lacks ecological validity. Therefore, new assessment methods are needed for the early identification of this cognitive impairment.

**Objective:**

This study aimed to compare the applicability and stability of an immersive virtual reality (VR) system developed to assess route learning between older adults with and without mild cognitive impairment (MCI).

**Methods:**

The study sample included 43 older adults: 22 without MCI and 23 with MCI. Applicability was assessed based on the recording of adverse events and the sense of presence reported through questionnaires. The Mann–Whitney U test was applied to compare the applicability of the Spatial Orientation in Immersive Virtual Environment Test (SOIVET)-Route task between older adults with and without MCI. Both short- and long-term stabilities of the task were evaluated using the intraclass correlation coefficient (ICC).

**Results:**

The mean age of participants was 71.4 years (SD=5.5). A minimum number of adverse events (mean=1.46; SD=2.11) and high levels of presence (mean=138.04; SD=14.80) were reported, and there was no difference between groups with and without MCI. A good to excellent correlation was found for short-term stability (CCI 0.78) and a reasonable correlation was found for long-term stability (CCI 0.58).

**Conclusions:**

The VR system was applicable for older adults and showed a good to excellent correlation for short-term stability.

## RESUMO.

Orientação espacial é a capacidade de encontrar um caminho em um ambiente, seguir rotas familiares, reconhecer lugares e aprender novas rotas. A desorientação espacial é um dos primeiros sintomas da doença de Alzheimer, e a avaliação cognitiva tradicional carece de validade ecológica. Diante disso, novos métodos de avaliação são necessários para a identificação precoce desse comprometimento cognitivo.

**Objetivo:**

Este estudo teve como objetivo comparar a aplicabilidade e a estabilidade de um sistema de realidade virtual imersivo desenvolvido para avaliar a aprendizagem de rotas entre idosos com e sem comprometimento cognitivo leve (CCL).

**Métodos:**

Participaram do estudo 43 idosos: 22 sem CCL e 23 com CCL. A aplicabilidade foi avaliada por meio do registro de eventos adversos e pela sensação de presença relatados. O teste de Mann-Whitney foi aplicado para comparar a aplicabilidade da tarefa SOIVET-*Route* entre idosos com e sem CCL. A estabilidade da tarefa em curto e longo prazo foi avaliada pelo coeficiente de correlação intraclasse (ICC).

**Resultados:**

A idade média dos participantes foi de 71,4 anos (desvio padrão — DP=5,5). Em relação à aplicabilidade, encontramos mínimo relato de sintomas adversos (média=1,46; DP=2,11) e altos níveis de sensação de presença (média 138,04; DP=14,80), e não houve diferença entre os grupos com e sem CCL. Ao analisarmos a estabilidade, encontramos de boa a excelente correlação em curto prazo (CCI=0,78) e uma correlação razoável em longo prazo (CCI=0,58).

**Conclusões:**

O sistema de realidade virtual foi aplicável em idosos e mostrou boa correlação na estabilidade de curto prazo.

## INTRODUCTION

Topographical orientation, or spatial orientation, is the ability to find one’s way around an environment, follow familiar routes, recognize places, and learn new routes, and it is an essential skill for a person’s autonomy^1,2^. The main spatial orientation strategies are egocentric and allocentric^3^.

Along with these strategies, the individual needs to remember a series of motion directions at decision points to walk on a path at a greater distance than can be viewed in a single time, in other words, the direction of the destination^1,4^. A sense of direction leads to learning a new route, where the subject, within the environment itself, incorporates information through repeated visualizations of the environment and continuous changes in the egocentric orientation, being able to, when walking this path again, go from your origin to your destination^1^.

Spatial disorientation is a common finding in Alzheimer’s disease (AD), even in its early stages and, since the neurodegenerative process of AD precedes the clinical signs for diagnosis, it is believed that the detection of spatial disorientation in patients with mild cognitive impairment (MCI) is a predictor of higher risk of conversion of these patients^5^. Thus, the assessment of spatial orientation associated with other clinical assessments in patients with MCI can be a cognitive marker of AD^6,7^.

There is no consensus on the best way to assess spatial orientation, and the traditionally used pen and paper tests are not sufficiently sensitive and ecological to detect spatial disorientation^6^. An assessment is considered ecological when it investigates the patient’s skills and difficulties as close as possible to their reality, exposing them to their daily life problems^8^. Non-ecological assessments do not adequately evaluate the impairment of spatial orientation experienced by the patient in the real world^9^.

New computerized methods have been developed to assess spatial orientation^10,11^. The use of virtual reality (VR) allows the patient to interact in environments like real ones through projection in three-dimensional (3D) scenarios and the use of one’s sensory channels to interact with the visual and auditory stimuli of virtual systems^6,12,13^. The sense of presence and immersion experienced by the patient in an immersive virtual environment is greater, and the individual can interact with its elements, favoring their behavior as if they were acting in the real world^14,15^. Thus, immersive VR could simulate more realistic environments when compared to paper and pencil assessments and, therefore, more accurately reproduce the difficulties of spatial orientation and identify more subtle deficits^15,16^.

With the lack of a reference standard to assess spatial orientation and the advantages of evaluating this cognitive domain through ecological tasks, our research group developed a system called Spatial Orientation in Immersive Virtual Environment Test (SOIVET)^7,17^. This system contains a task called Route (SOIVET-Route), which focuses on route learning.

In 2021, Costa et al.^18^ analyzed the concurrent validity of the SOIVET-Route, concluding that it is a valid tool for assessing the spatial orientation of older adults, but its applicability and stability have not yet been analyzed. Therefore, this study aimed to compare the applicability of the SOIVET-Route in older adults with and without MCI. Besides, we assessed the short- and long-term stability of its assessments.

## METHODS

### Study sample

This study was conducted by following the guidelines and regulatory standards for research involving human beings (resolution 466/12 of the National Health Council), consubstantiated opinion no. 2.580.187, with the Certificate of Presentation for Ethical Appreciation (CAAE) no. 84904018.6.0000.0065, approved by the Ethics and Research Committee of the Faculdade de Medicina, Universidade de São Paulo.

Data collection started in February 2019 and ended in March 2020 involving the participation of 45 older adults: 22 were elderly residents in the community without objective evidence of cognitive impairment verified through a screening test – the Addenbrooke’s Cognitive Examination – Revised (ACE-R)^19^ and 23 elderly individuals with a diagnosis of MCI referred from the Hospital das Clínicas of the Faculdade de Medicina of the Universidade de São Paulo.

Eligibility criteria included age ≥60 years; absence of a history of vertigo and/or labyrinthopathy; normal or corrected visual and auditory acuity; written informed consent form (ICF) for participation in the study; a score of >82 on the ACE-R for the control group; and a diagnosis of MCI according to Petersen’s criteria^20^ for the MCI group. Participants with incapacity to understand the instructions and interact with the tasks were excluded. In addition, participants who presented any limiting adverse events during the experience with the immersive system were excluded. Finally, older adults who abandoned the study before completing all its stages were excluded (dropouts).

### Data collection

The project was presented to the participants who met the eligibility criteria of the study. After agreeing to participate and signing the ICF, the participants were assessed in two different moments with an interval of 7–14 days between the sessions.

In the first moment, data collection started with the application of the ACE-R. Later, the sociodemographic characterization, motion sickness screening, and technology use profile questionnaires were applied.

After completing the questionnaires, the participants were guided to perform the tasks. Finally, immediately after completing the VR task, the Witmer and Singer Presence Questionnaire^21^ and the questionnaire to identify adverse symptoms were applied.

In the second moment, only the task in a virtual environment was reapplied.

### Research procedures

#### Sample characterization, cognitive screening, and motion sickness screening

A sociodemographic questionnaire and a questionnaire describing familiarization with the use of technology, both developed by the study authors, were applied to characterize the sample^7^. Scoring on the technology use profile questionnaire varies from 0 to 40 points; with the higher the score, the greater the familiarity of participants with technology.

The ACE-R was applied for cognitive screening. Individuals with ACE-R score of >82 were included in the group of older adults without cognitive impairment. This score is considered a cutoff value for older adults with cognitive impairment without dementia^22^.

The absence of a history of vertigo and labyrinthopathy was assessed through self-report. Participants were also assessed using a motion sickness screening questionnaire, prepared by the study authors, with scores ranging from 0 to 6 points, with the higher the score, the greater the discomfort prior to task performance.

#### SOIVET-Route task

The task performed is an adaptation of the Route subitem of the Rivermead Behavioral Memory Test (RBMT)^23^. The test can be conducted in a wide variety of locations and does not establish a minimum or maximum distance between them.

**Figure 1:**
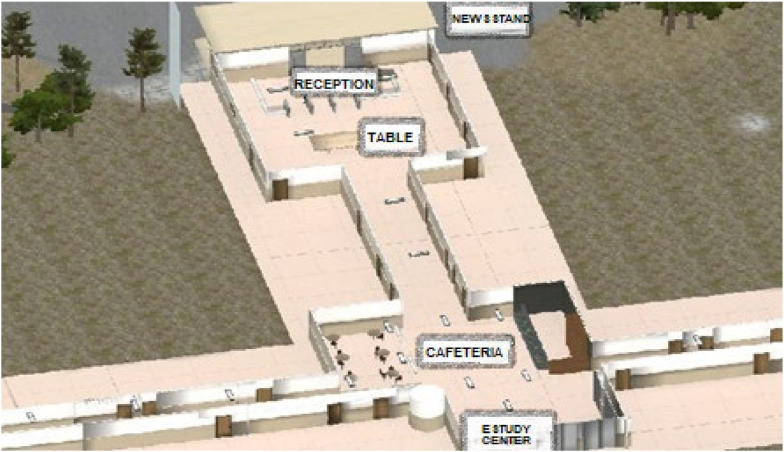
Route in the real and virtual tasks (aerial view of the virtual task).

**Figure 2:**
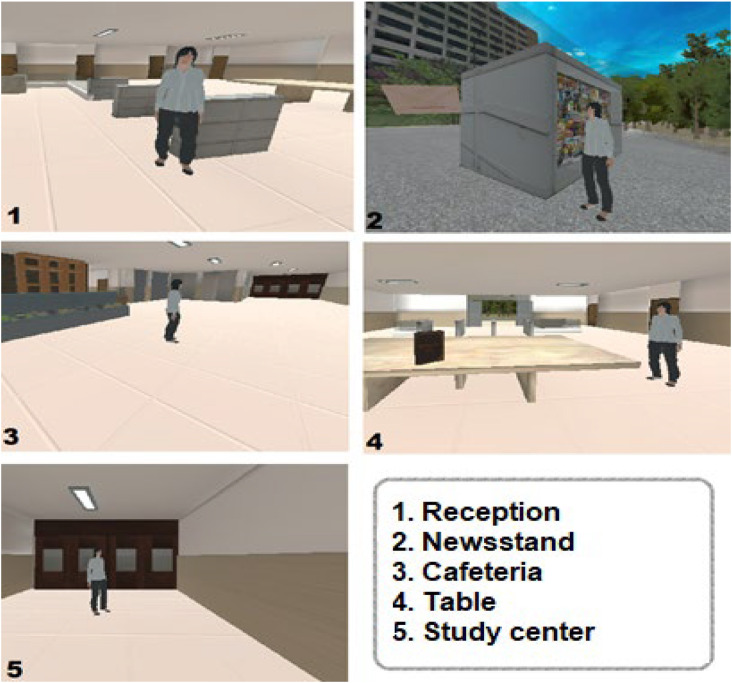
Reproduction of the real environment in virtual reality.

In this study, we used the main entrance of the Hospital das Clínicas of the Faculdade de Medicina of the Universidade de São Paulo (ICHC-FMUSP) as a reference for the development of the task in a virtual environment, using similar dimensions to simulate the virtual environment task as realistically as possible, and it occurred in the following four stages:

- The avatar took the route together with the participant stopping at five different places with time to observe the surroundings. The stop points were the reception, a newsstand outside the building, a cafeteria, a table, and the entrance to the study center in this order (Figure 1).
- After completing the route with the avatar, the participants were invited to retrace the route alone stopping at the same places (immediate recall task).
- After 20 min, the individual was asked to recreate the route once again (late recall task).
- The participants were invited to return to the data collection place between 7 and 14 days to perform the abovementioned items 1–3 (Figure 2).

The system was developed using the Oculus Rift® head-mounted display and its controller.

The system automatically extracts the number of locations visited in the correct (correct) or incorrect (errors) sequence. The score, which can vary from 0 to 5 points, is assigned according to the participant’s performance and the number of correct answers (places visited in the correct sequence).

#### Assessment of the system applicability in older adults with and without mild cognitive impairment

Applicability was evaluated based on the report of adverse symptoms triggered by the task, such as general discomfort, headache, nausea, pallor, vomiting, sweating, and fatigue. The occurrence of these symptoms was assessed through a self-report questionnaire in which the higher the score, the lower the tolerability of the task performed, with a maximum score of 64 points^7^.

The sense of presence was assessed using the Witmer and Singer Presence Questionnaire^21^. This questionnaire aims to measure the degree that individuals experience immersion in an environment, depending on the attentional resources existing in the environment to be explored. The questionnaire comprises 22 items, totaling 154 points. In the end, the higher the score, the greater the sense of presence and immersion, that is, the task applicability.

#### Assessment of short- and long-term stability

Stability is the degree to which similar results are obtained at two different times, and for that, two correlations were made between the participant’s performance in the SOIVET-Route task at two different times: correlation between immediate recall and late recall (short-term stability) and correlation between the first and second test days (long-term stability).

### Statistical analysis

The collected data were processed using the SPSS version 20.0 software. The participants’ clinical and sociodemographic characteristics were expressed as mean, standard deviation (SD), and 95% confidence interval (95%CI) for the numerical variables, whereas the categorical variables were expressed as absolute and relative frequencies.

Short- and long-term stabilities were analyzed using the intraclass correlation coefficient (ICC) between the first and second evaluation days and between the immediate and late recall evaluations performed on the same day using the following analysis criteria: ICC<0.4: poor, 0.4–6: reasonable, 0.6–0.75: good, and 0.75–1.0: excellent. We adopted an alpha of 0.05 as statistical significance^24^.

The Mann-Whitney U test was applied to compare the applicability measures of the SOIVET-Route task between older adults with and without MCI. Hedge’s g test was used to calculate the effect size considering the following results: large ≥0.8, medium 0.8–0.2, and small <0.2^25^.

## RESULTS

The mean age of participants was 71.4 years (SD=5.5), and there was a predominance of females (n=28; 62%). Most participants had completed their higher education (n=29; 65%) and reported having some comorbidity (n=33; 75%). The most common comorbidities were systemic arterial hypertension, diabetes mellitus, and osteoporosis.

Among the 45 older adults who participated in the study, 23 (51%) met MCI diagnostic criteria and the other participants presented no complaints or evidence of having cognitive impairment, which verified through higher scores on the ACE-R, with a statistically significant difference in relation to those with MCI (p<0.001) (Table 1).

**Table 1:** Characterization of the total elderly sample and comparison between older adults with and without mild cognitive impairment.

		Total sample (n=45)	Elderly people without MCI (n=22)	Elderly people with MCI (n=23)	p-value
Age (years)		71.4±5.5	71.0±5.7	71.8±5.4	0.53^a^
Gender, n (%)	Female	28 (62)	14 (64)	14 (61)	1.00^c^
	Male	17 (37)	8 (36)	9 (39)	
Education, n (%)	First grade completed	2 (4)	2 (9)	0 (0)	0.005^c^
	Second grade completed	12 (27)	2 (9)	10 (43)	
	Higher education	29 (65)	18 (82)	11 (48)	
	Graduate course	2 (4)	0 (0)	2 (9)	
ACE-R		90.5±6.3	94.9±2.8	86.3±5.8	<0.001^b^
Motion sickness screening		0.55±1.13	0.40±1.18	0.69±1.10	0.08^a^
Familiarity with the technology		17.8±8.4	20.4±8.3	15.3±8.0	0.034^a^
Comorbidities, n (%)	Yes	33 (75)	17 (77)	16 (73)	1.00^c^
	No	12 (25)	5 (23)	6 (27)	

Numerical values represented by average±standard deviation and absolute number (%).

a p-value referring to the Mann-Whitney U test;

b p-value referring to the Student’s *t*-test for independent samples;

c p-value referring to Fisher’s exact test. MCI: mild cognitive impairment; ACE-R: Addenbrooke’s Cognitive Examination – Revised.

### Assessment of applicability

The elderly participants reported a small number of adverse events (mean=1.46; SD=2.11), with mild dizziness and nausea as the most frequent, and high levels of sense of presence and immersion (mean=138.04; SD=14.80). There was no difference between groups with and without MCI (Table 2).

**Table 2:** Tolerability and sense of presence of the SOIVET-Route in the total sample of older adults and comparison between older adults with and without mild cognitive impairment.

	Total sample (n=45)	Elderly people without MCI (n=22)	Elderly people with MCI (n=23)	p-value	Hedge’s g
Cybersickness	1.46±2.11	1.13±1.93	1.78±2.27	0.29	0.30
Sense of presence and immersion	138.04±14.80	137.81±14.62	138.26±15.30	0.73	0.03

Numerical values represented by average±standard deviation; p-value referring to the Mann-Whitney U test; effect size (Hedge’s g): large ≥0.8, medium 0.8–0.2, small <0.2. MCI: mild cognitive impairment; D1: day 1; D2: day 2.

However, 17% of participants were unable to complete the task due to complaints of nausea and severe dizziness: 5 (23%) elderly people without MCI and 4 (17%) with MCI. These participants were not included in the analyses, keeping 45 elderly graduating.

There was no correlation between the participants’ scores in the questionnaires of adverse symptoms or sense of presence with age, gender, education, screening for motion sickness, and technology usage profile.

### Assessment of short- and long-term stability

Assessment of short- and long-term stability showed an excellent correlation for short-term stability (immediate and late recall, performed with an interval of 20 min) with effect size of 80% power, but poor to reasonable correlation for long-term stability (performed with an interval of 7–14 days) (Table 3).

**Table 3:** Intraclass correlation coefficients between the first and second assessment days and between immediate and late recall performed on the same day of the SOIVET-Route task.

	ICC D1×D2		ICC Immediate×late	
Total sample (n=45)	Immediate recall (virtual)	0.58^b^	D1	0.78^b^
	Late recall (virtual)	0.37^a^	D2	0.81^b^
Elderly people without MCI (n=22)	Immediate recall (virtual)	0.28	D1	0.60^b^
	Late recall (virtual)	0.03	D2	0.73^b^
Elderly people with MCI (n=23)	Immediate recall (virtual)	0.63^b^	D1	0.80^b^
	Late recall (virtual)	0.44	D2	0.85^b^

ICC<0.4: poor; 0.4–0.6: reasonable; 0.6–0.75: good; 0.75–1.0: excellent; effect size with 80% power: ICC≥0.5. ICC: intraclass correlation coefficient; MCI: mild cognitive impairment; D1: day 1; D2: day 2

a p<0.05

b p≤0.01.

## DISCUSSION

This study analyzed the applicability of the SOIVET-Route task and its respective stability in older adults with and without MCI.

For a VR assessment to be applicable in the elderly population, a low rate of adverse symptoms is necessary^15,26^. In this study, the elderly participants had good tolerability to perform the task, and there were minimal reports of adverse symptoms among those who managed to complete the assessment. There was no difference between groups and the incidence of dropouts because tolerability was 17%, which is considered low compared with the rates of 30–80% found in the literature^27^. There was also no correlation between the scores of the adverse symptoms’ questionnaire and age, gender, education level, or familiarity with technology. This indicates that these factors did not influence the applicability of immersive VR in the elderly population. Nevertheless, the immersive system needs to be accurate in capturing the patient’s movements and the lowest latency possible in relation to the image displacement so that there is no sensory conflict and occurrence of possible adverse events^15,27^. These findings corroborate the study conducted by Kim et al.^15^, who used the Oculus Rift® to assess elderly people with and without Parkinson’s disease and obtained the results similar to this research. The studies that identified higher incidence rates of adverse events used immersive VR devices with lower visual processing speed, which induced delays between movement and simulation, increasing the occurrence of possible adverse symptoms.

The sense of presence experienced by the patients was another important aspect of the applicability of immersive VR. The greater the sense of presence in a VR task, the more the individuals experience actions and emotions similar to real-life situations, making it more environmentally friendly^7,28^. In this study, the high sense of presence scores were verified, and there was no statistically significant difference between the groups, suggesting that the SOIVET-Route task can be an ecological task despite having fewer sensory cues compared with the same task performed in a real environment.

Regarding the short- and long-term stability of the SOIVET-Route task, the analysis showed a good to excellent correlation in short-term stability (immediate and late recall, performed with a 20-min interval) and a poor to reasonable correlation in long-term stability (between the first and second test days, performed with an interval of 7–14 days). Bearing in mind that the system itself provides the orientation regarding the proposed activity during the task and registers the score, no performance bias from the evaluators is expected. This difference in short- and long-term stability could be explained by improved performance as a result of practice, as the participants were repeatedly exposed to the task, especially in the group of elderly people with MCI.

Among the studies that have investigated spatial orientation in elderly people through VR^10,29–34^, only Pouya et al.^32^ addressed test stability. Pouya et al.^32^ obtained strong correlations in their analyses, but the participants repeated the VR assessment after 6 and 12 months of the first assessment, which may have prevented a possible learning effect and/or memorization of the task because of the long gap between exposures.

To better generalize the results, considering that the elderly performed better as they were repeatedly exposed to the task, it would be interesting to have more time for them to become familiarized with the system before recording their performance. A variation in the location of the five stop points during the route—in case there was a need to reapply the task at shorter intervals—would be interesting to avoid possible route memorization.

Finally, transposition of the immersive VR task to a tablet with a rotation sensor could be an alternative for the elderly people who presented adverse symptoms in immersion. However, the task would most likely have a lower sense of presence compared with immersive VR, but it would still be advantageous for elderly people with greater sensitivity to motion sickness.

The VR system developed to assess the route learning was applicable for older adults with and without MCI. The assessments showed good short-term stability. These results encourage the use of innovative tasks and immersive virtual environments for the assessment of cognition in older adults.
